# Chinese immigrant parents’ vaccination decision making for children: a qualitative analysis

**DOI:** 10.1186/1471-2458-14-133

**Published:** 2014-02-07

**Authors:** Linda DL Wang, Wendy WT Lam, Joseph T Wu, Qiuyan Liao, Richard Fielding

**Affiliations:** 1 Health Behaviour Research Group, Division of Behavioural Health, School of Public Health, The University of Hong Kong, 5/F William Mong Block, 21 Sassoon Road, Pokfulam, Hong Kong; 2 Division of Epidemiology and Biostatistics, School of Public Health, The University of Hong Kong, 5/F William Mong Block, 21 Sassoon Road, Pokfulam, Hong Kong

**Keywords:** Vaccination decision making, Social norm, Immigrants, Qualitative analysis, Grounded theory

## Abstract

**Background:**

While immunization coverage rates for childhood routine vaccines in Hong Kong are almost 100%, the uptake rates of optional vaccines remain suboptimal. Understanding parental decision-making for children’s vaccination is important, particularly among minority groups who are most vulnerable and underserved. This study explored how a subsample of new immigrant mothers from mainland China, a rapidly-growing subpopulation in Hong Kong, made decisions on various childhood and adolescent vaccines for their offspring, and identified key influences affecting their decision making.

**Methods:**

Semi-structured in-depth interviews were conducted with 23 Chinese new immigrant mothers recruited by purposive sampling. All interviews were audio-taped, transcribed and analyzed using a Grounded Theory approach.

**Results:**

Participants’ conversation revealed five underlying themes which influenced parents’ vaccination decision-making: (1) Institutional factors, (2) Insufficient vaccination knowledge and advice, (3) Affective impacts on motivation, (4) Vaccination barriers, and (5) Social influences. The role of social norms appeared overwhelmingly salient influencing parents’ vaccination decision making. Institutional factors shaped parent’s perceptions of vaccination necessity. Fear of vaccine-targeted diseases was a key motivating factor for parents adopting vaccination. Insufficient knowledge about vaccines and targeted diseases, lack of advice from health professionals and, if provided, suspicions regarding the motivations for such advice were common issues. Vaccination cost was a major barrier for many new immigrant parents.

**Conclusions:**

Social norms play a key role influencing parental vaccination decision-making. Insight gained from this study will help inform healthcare providers in vaccination communication and policymakers in future vaccination programme.

## Background

Vaccination is among the most successful and cost-effective public health strategies for controlling a variety of communicable diseases [1]. In Hong Kong, childhood vaccines are administered under two strategies. Most routine vaccines (B.C.G, Hepatitis B, Diphtheria, Pertussis and Tetanus (DPT), Polio, Pneumococcal, and Measles, Mumps & Rubella (MMR)), mandated under government’s Childhood Immunization Programme (CIP) are free to all local-born and resident children and administered according to standard schedules from birth in hospitals, throughout pre-school via the Department of Health (DH) Maternal and Child Health Centres (MCHCs), and into primary school years by DH outreach School Immunization Teams (SITs). Conversely, optional vaccines including Varicella, *Haemophilus* influenza type b, seasonal influenza A, Hepatitis A, Japanese encephalitis, Rotavirus, Meningococcal, and Human Papillomavirus (HPV) vaccines, are administrated on voluntary basis via primary care clinics the costs of which are fully or partially borne by recipients. Hong Kong has almost universal immunization coverage rates for mandated vaccines of 98% or above among local-born children and over 95% among Mainland-born children living in Hong Kong [2]. These rates are higher than reported in many developed countries/regions [3,4]. For instance, in 2010 only 72.7% of 19–35 month old children received all six USA government-recommended vaccines [4]. However, in Hong Kong optional childhood vaccines have much lower uptake rates, being 32% and 15%, respectively among local-born 2-5-year-old children for varicella and seasonal influenza vaccination respectively, the most frequently adopted optional childhood vaccines [2].

Despite Severe Acute Respiratory Syndrome (SARS) and the 2009 pandemic influenza (pA/H1N1) focusing attention on vaccination and how best to protect individuals during epidemics and/or pandemics [5], the actual uptake rate of novel 2009 pA/H1N1 influenza vaccine was only 1.1% among the Hong Kong general public [6], while no data are available about the pA/H1N1 vaccination rate among children. Increasingly microbial factors are implicated in noncommunicable diseases (NCDs) indicating growing possibilities for vaccination against adulthood NCDs [7]. The recently introduced HPV vaccine is a typical example targeting young adolescent girls to prevent adult-onset cervical cancer [8]. Nevertheless, since its 2006 launch in Hong Kong, only 2.4% of secondary school girls [9] have been vaccinated against HPV, compared to western countries where uptakes range between 17-81% [10].

Parents mostly control young children’s access to vaccines, so understanding parental decision-making for their children’s vaccinations (VDM) is important. An extensive literature has identified factors promoting or inhibiting parents’ vaccination acceptance and decision-making. Principal influences are perceived severity and susceptibility of vaccine-preventable diseases (VPDs), beliefs about efficacy and safety of vaccines, and the cost of vaccination [11-15]. Additionally, social context, medical authorities (government, family doctors) and peers may also inform parental notions and attitudes about vaccines and VPDs related-risk [12,15-20]. However, most existing studies are quantitative studies focusing on single vaccines, which limit understanding of how parental vaccination values and beliefs were shaped and interact, translating into vaccination decisions. Moreover, only fewer studies have involved minority groups in a community regarding parental VDM for children [21,22]. No study we could find has combined all three of these features targeting Chinese migrants.

Annually around 50,000 new immigrants from mainland China settle in Hong Kong presenting the most rapidly-growing subpopulation. Half of the migrants are adults, 90% of whom are female. Within Chinese culture, mothers remain the main caretakers of children and in most Hong Kong households make the family healthcare decisions, including vaccination decisions regarding their children [23]. Therefore, our study focused on new immigrant mothers.

This paper describes a qualitative study of new immigrant mothers from mainland China living in Hong Kong and examines the issues behind parental VDM to protect children’s current and future health.

## Methods

### Study design and sample

A qualitative study with individual in-depth interviews under a Grounded Theory approach was chosen because it allows for unconstrained study of the range and experiential aspects of target perceptions, behaviours and underlying issues [24,25]. It attempts to avoid presumptions, thereby enabling a broad-brush picture of key concerns to emerge as the ground for theory building. It is most useful when either little is known, or there is a wish to minimize presumptions about the target behaviours.

Ethnic Chinese women who migrated from Mainland China (a majority from Guangdong Province) to Hong Kong no more than 7 years ago (the minimum eligibility period for Hong Kong Special Administrative Region permanent residency), and have at least one child aged 14 years or younger living in a Hong Kong household were eligible for this study. These inclusion criteria gave an initial starting point for data collection. Participants were recruited using purposive sampling, where respondents meeting heterogeneous socio-demographic criteria are targeted to capture the richness of the phenomenon. Friends and acquaintances referred by the original interviewees where different beliefs and/or behavior/practice were noticed were also purposively included to help capture maximum diversity of opinion. Under Grounded Theory, data collection and data analysis run parallel in an iterative process. Using insights gained from ongoing data analysis, participants were further chosen based on particular characteristics, such as age, socio-economic background, education level, and children’s vaccination status, in order to achieve diversity and enhance understanding of various facets of the phenomenon being studied. Sample size was determined by data saturation (no new material emerging over three consecutive interviews).

### Data collection

This study was approved by the Institutional Review Board of the University of Hong Kong/Hospital Authority, Hong Kong West Cluster. All participants were informed by the interviewer (LDLW, a female native Mandarin/Putonghua speaker from mainland China) about the study purpose and procedures, and right of uncontested withdrawal. After giving written informed consent, semi-structured individual in-depth interviews were conducted and digitally recorded. Interview locations were determined by participants for their privacy and convenience.

Participants were initially asked whether their children had received all or some routine vaccines recommended by Hong Kong or mainland China governments, and to elaborate their choices. Participants were encouraged to discuss their attitudes and concerns regarding vaccination for their child(ren), and their sources of vaccination information. To improve insight into their attitudes and VDM, mothers were asked about various types of routine and optional vaccines including those for preventing childhood diseases (chickenpox and seasonal influenza), novel vaccines (2009 pA/H1N1 influenza), and HPV vaccine for adult-onset cancer prevention. The reasons why they decided for or against their children receiving a vaccine were explored using questions and prompts to encourage response elaboration. All interviews were conducted in Putonghua. Any medical concepts (such as pA/H1N1, HPV) were introduced in everyday language commonly used in local mass media.

### Data analysis

Interviews were performed concurrently with transcript analysis using constant comparative methods under Grounded Theory [24] to explore emergent themes in subsequent interviews. Data analysis under Grounded Theory has three coding stages. Data were first broken down by open coding whereby each event, idea or other element pertaining to a phenomenon, in each line of every transcript was labeled. Similar concepts were grouped and named into one category. Next axial coding was used to explore interrelationships between categories. Coded categories were specified into subcategories by identifying causal conditions, contexts, actions or consequences to build interconnections between categories. Finally, selective coding identified core categories associated with the research questions and their relationships with other categories wherein relevant findings of research interest were integrated and refined [24]. To maximize analytic validity, two investigators (LDLW and WWTL) independently coded the data and held joint interpretive discussions. Disagreements were resolved by repeated textual reference, comparison and discussion, and, where necessary hierarchy re-assembly and re-coding. QSR NVivo 10 was used to facilitate the analytic process. Data analysis was based on original verbatim transcripts written in Chinese. The quotation in the Results section were presented in English after having been translated and back-translated using ethnographic principles to ensure equivalent meanings.

## Results

Twenty-three new immigrant mothers participated between October 2011 to May 2012. Interviews lasted between ~30 to 80 minutes. Participants’ ages ranged from 27 to 50 years old (median 34 years). Fifteen (65%) participants were educated to secondary level, approximating to the 60% proportion of female new immigrants to Hong Kong aged 15+ achieving lower secondary as their highest education level [26]. Sixteen participants had monthly family incomes below HK$14,070 (~US$1,800), the median monthly domestic income of new immigrant households in 2011 [26]. Most participants were One-way Permit Holders who came to Hong Kong to join their husbands, a profile comparable to the general picture of female new immigrants in the 25-44 age group [26] (Table 1).

**Table 1:** Characteristics of the participants

**Code**	**Age (y)**	**Marital status**	**Birth place (Province)**	**Children**’**s age range (m, y)**	**Occupation**	**Educational attainment**	**Family income (HKD)**
IM 1	36	Married	Guangdong	5 y	Housewife	Upper secondary	~4,000
IM 2	33	Separated	Guangdong	4 y	None	No schooling	~3,000
IM 3	34	Married	Hunan	2 y	Housewife	Lower secondary	17,000
IM 4	43	Married	Jiangxi	14-15 y	None	Primary	~6,000
IM 5	38	Married	Guangdong	7-9 y	Self-employed	Upper secondary	17,000-20,000
IM 6	33	Married	Sichuan	7 y	Part-time job	Lower secondary	10,000-15,000
IM 7	34	Married	Hubei	10 m-9 y	Housewife	Upper secondary	30,000-40,000
IM 8	44	Widow	Jilin	7 y	None	Lower secondary	4,000-5,000
IM 9	34	Married	Guangdong	5-10 y	Housewife	Upper secondary	~10,000
IM 10	41	Married	Guangdong	6-8 y	None	Primary	13,000
IM 11	34	Married	Guangdong	6 y	Part-time job	Post-secondary	~10,000
IM 12	50	Married	Jilin	13-23 y	Housewife	university	>30,000
IM 13	32	Married	Guangdong	8 m-9 y	Housewife	Lower secondary	~20,000
IM 14	32	Married	Guangdong	2-8 y	Housewife	Primary	~12,000
IM 15	33	Married	Guangdong	4-8 y	Housewife	Lower secondary	5,000-6,000
IM 16	39	Married	Guangdong	2-6 y	Housewife	Primary	8,000
IM 17	38	Married	Guangdong	1.5-13 y	Housewife	Upper secondary	~8,000
IM 18	32	Married	Guangdong	8 m-6 y	Housewife	Lower secondary	9,200-9,800
IM 19	38	Married	Hunan	1.5-16 y	Housewife	Primary	~7,000
IM 20	29	Married	Guangdong	1.5-5 y	Housewife	Primary	~13,000
IM 21	27	Married	Guangdong	1 m-5 y	Housewife	Primary	~8,200
IM 22	31	Married	Guangdong	4 y	Housewife	Lower secondary	~12,000
IM 23	36	Married	Jilin	10 y	Housewife	Upper secondary	20,000

**Figure 1:**
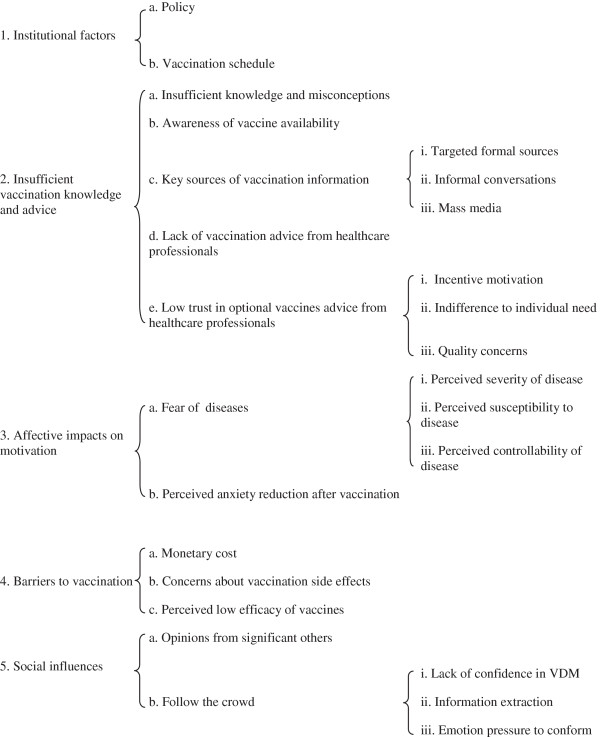
Hierarchical chart of themes and categories.

Overall, five major themes addressing parental VDM emerged: (1) Institutional factors, (2) Insufficient vaccination knowledge and advice, (3) Affective impacts on motivation, (4) Vaccination barriers, and (5) Social influences. Figure 1 depicts relationships between themes and categories. Because the study is designed to identify the spectrum of opinion, and not prevalence of opinion, the precise numbers and proportion of participants expressing each view are not provided, as these data are not meaningful in a non-randomized small qualitative study such as this. Excerpts from different respondents are used to illustrate the themes, categories and elements that emerged. They in no way reflect frequency of expressed opinion. For those readers curious about proportions of responses, we used the terms “few” for under five respondents, “several” for between 5-10, “majority” for 11-15, and “most” for 16-23.

### Institutional factors

Institutional factors capture vaccination-related factors influencing parents’ VDM stemming from the healthcare system, including policies and vaccination schedules.

a. Policy

All participants were receptive to all routine vaccines mandated by either Hong Kong or mainland China governments. Because mainland China has had a childhood vaccination policy for several decades, study participants expressed unanimous confidence about the safety, efficacy and necessity of routine vaccinations in Hong Kong.

*Because it has been said for many years that children should be vaccinated*, *I was also vaccinated when I was a kid. Since it*’*s already been used for so many years*, *it should be good*. (*IM8*)

*Those vaccines* (*are*) *being requested by government*, (*so*) *I*’*m not concerned* (*about vaccinations*). (*IM19*)

In contrast, a minority of participating mothers had decided to reject all optional vaccines as unnecessary since they were not formally recommended by government agencies.

*I don*’*t think they* (*optional vaccines*) *are so important. If they are really important*, *usually the government will ask you to be vaccinated against it*. (*IM15*)

School admission obliges children to be compliant with CIP coverage. This was a key driver for almost all participants. Similar to studies elsewhere [27], vaccines mandated for school entry strengthen parents’ perception that every child has to be vaccinated, which also somewhat mitigates parents’ concerns about vaccine safety.

*Those* (*vaccines*) *are mandatory. You have to have the shot*, *otherwise*, *when kids are admitted to any kindergarten or school*, *they will check the vaccination record. They won*’*t accept* (*your child*) *if you are not vaccinated. It is the pressure*. (*IM8*)

*I don*’*t worry about it* (*side*-*effect*), *because every child is vaccinated in this way*, *no need to worry*. (*IM21*)

b. Vaccination schedule

The explicit schedule for routine vaccination was considered important, driving parents to fulfill the social contract.

*When she was born*, *they gave* (*us*) *a record card. They have arranged all the schedules of each* (*routine*) *vaccine. When you go for injection*, *they would make appointment for the next shot*. (*IM8*)

Conversely, for many optional vaccines, for example HPV vaccine, the timing of vaccination was perceived by a majority of participant mothers as much more fluid and uncertain. In Hong Kong, the quadrivalent HPV vaccine is approved for 9-45-year-old women and the bivalent for women aged 10-45 years. This wide eligibility period confused some parents about the appropriate age for vaccination.

*It*’*s said 9 years old or later. But*, *when exactly is the appropriate age to vaccinate*? (*IM8*)

*Ah*, *I heard that girls aged 15*-*16 years can take the vaccination*, *14 year*-*old is also ok*, *or women already married*, *having sexual experience*, *or suspecting of having this disease* (*cervical cancer*), *all can be vaccinated* (*against HPV*). (*IM3*)

### Insufficient vaccination knowledge and advice

Knowledge-related deficits were widespread and varied, and included misconceptions, ignorance and procedural uncertainties. Formal (for example DH websites) and informal (for example friends) information sources were used.

a. Insufficient knowledge and misconceptions

Participants had poor knowledge and comprehension regarding VPDs, how vaccines work, and their administration. Although insufficient knowledge did not apparently hinder parents’ acceptance of routine childhood vaccination, ignorance surrounding optional, particularly newly introduced vaccines, is undesirable.

…*just like diphtheria. Um*, *I have no idea about diphtheria. I really do not know what it is. But it is compulsory*, *so I took her for injection*. (*IM22*)

*It* (*cervical cancer*) *is because of promiscuity*, *isn*’*t it*? *But I watched from TV that she* (*the character of a TV program*) *only had sexual relationship with her husband but she also suffered cervical cancer. I have no idea why it happened*. (*IM22*)

*What I want to know* (*about HPV vaccine*) *is where the injection will be given. Will it be a shot into her uterus or somewhere else*? *I have no idea*. (*IM15*)

b. Awareness of vaccine availability

Some respondents were unaware that vaccines are available for many common childhood diseases. Chickenpox vaccine, though available since 1995, was frequently mentioned.

*There*’*s a vaccine for Chickenpox*? *I*’*ve never heard of it*. (*IM14*)

*I know chickenpox*, *but I don*’*t know there is (a) chickenpox vaccine*. (*IM21*)

Despite government efforts in 2009-2010 to promote vaccination for children against 2009 pA/H1N1 influenza given the extremely low vaccination uptake, several mothers interviewed here were surprisingly unaware that the vaccine was available during the pandemic, despite already living in Hong Kong.

*No*, *I haven*’*t heard of swine flu vaccine. Did it exist*? *I don*’*t know. No. In 2009 my daughter was around 5 years old. Um*, *I didn*’*t hear of it*. (*IM8*)

Moreover, as the child gets older, following the CIP regimen of single and combination vaccines confused a few participant mothers about which vaccines had already been given and which not. So a proportion of these parents may fail to make informed decisions and some missed the opportunity for vaccination.

*I thought the government had provided it* (*chickenpox vaccine*)…*Until she had chickenpox and went to see doctor. I heard that the vaccine was not in the government list and realized she hasn*’*t been vaccinated*. (*IM22*)

c. Key sources of vaccination information

The most commonly reported sources of vaccination information were targeted formal sources, informal conversations with peers, and mass media.

i. Targeted formal sources

Formal sources mainly comprised those from healthcare workers regarding routine vaccines, such as vaccination record cards for new-born babies from hospitals, and pamphlets from MCHCs. Leaflets from children’s schools also worked as reminders, or an indicator of “official” promotion, prompting parents to vaccinate children.

*The school will send a leaflet to parents* (*about the seasonal influenza vaccination*)…*listing the costs from different private clinics in the district*, *you can choose it yourself*. (*IM11*)

*It felt like we weren*’*t encouraged to take Swine flu* (*vaccine*). *(My) daughter*’*s school didn*’*t ask for vaccination*, *the leaflet from school also didn*’*t mention it*. (*IM17*)

ii. Informal conversations

For all participants, conversations, particularly among peers or fellow parents, were salient sources of information and consultation about optional vaccines.

*Ask friends*, *those who are mothers too* (*are*) *experienced. Ask them whether they vaccinated their children and* (*if there was*) *any reaction after vaccination*. (*IM5*)

iii. Mass media

Television, newspapers, and internet (though several participants had access and necessary skills) were important sources of vaccination information. These sources not only promoted vaccination, but also provided adverse event reporting, apparently a more influential factor in parents’ acceptance of specific vaccines, particularly novel influenza pandemic vaccine.

*I heard of it* (*HPV vaccine*) *from television. It often has commercials* (*about it*). (*IM20*)

*In fact*, *initially we also wanted to vaccinate*, *because it* (*pA*/*H1N1 flu vaccine*) *was free for young children. But*, *later on the TV news reported*, *ah*, *what happened to the recipients. It was scary. Actually*, *I think TV is very influential. Once the TV reports negative news*, *because everyone would watch news*, *they will be scared*…*Ah*, *what if I*’*m unlucky*? *Better not to vaccinate*. (*IM11*)

d. Lack of vaccination advice from healthcare professionals

Similarly, several respondents reported ever receiving advice on optional vaccines from healthcare professionals. Some respondents also cited business-related explanations for why doctors seldom promote vaccination. Most attributed this to healthcare professionals’ lack of time, and avoidance of responsibility.

*If he* (*doctor*) *recommends and you take the vaccination*, *he will lose much business. This is my view. If everyone gets vaccinated*, *the clinic will lose a lot of business*. (*IM7*)

*I don*’*t think doctors dare to give you concrete advice*, *he cannot guarantee. He will ask you to decide yourself. If he recommends it*, *he has to take the responsibility. They are very clever*. (*IM11*)

e. Low trust in optional vaccines advice from healthcare professionals

Suspicions about motives were also raised in regard to doctors’ recommendations to take vaccination. Again financial gain was seen as a covert factor possibly influencing recommendations.

i. Incentive motivation

A majority of participants distrusted advice about optional vaccines from healthcare professionals, doubting their motivation.

*If I never heard of the vaccine*, *had no idea about it*, *(and) if he* (*doctor*) *recommends it to me*, *definitely I won*’*t accept it. Feel like it is a means of marketing*. (*IM6*)

ii. Indifference to individual need

A few participants felt that healthcare professionals recommended vaccines without carefully considering individual circumstances and needs, thereby discouraging parents who considered seeking vaccination advice from healthcare professionals.

*Regarding vaccines*, *I don*’*t trust doctors so much. Doctors will always say it is good*, *necessary to get vaccinated. I don*’*t trust doctors. I have my own judgment*. (*IM6*)

iii. Quality concerns

Compared to doctors in public healthcare settings, the quality of vaccines and safety of vaccination offered by private doctors were viewed with suspicion by a few participants, possibly because of reliability issues with some private clinics in Mainland China.

*If the doctor is from a government hospital*, *it is OK. If from the private sector*, *I would worry. Because*, *anyway*, *I trust government*’*s* (*recommendation*) *more*; *more guarantees. Vaccines from the private sector*? *I will be concerned about the safety*, *the quality issue*. (*IM14*)

### Affective impacts on motivation

Affect featured prominently in vaccination decision-making. Fear of the disease was balanced against possible reduction in worries after vaccination versus fearing the risk of vaccine-related harms.

a. Fear of diseases

Among participants who vaccinated or intended to vaccinate their children with optional vaccines, anxiety was expressed about diseases perceived as severe and uncontrollable, to which their child was felt to be susceptible.

i. Perceived severity of disease

Most participants believed that the mandated-vaccine preventable diseases were serious and justified vaccination.

*These vaccines are for preventing very serious illnesses*. (*IM13*)

Regarding optional vaccines, fear of the vaccine-targeted disease was a key factor motivating parents to adopt vaccination for prevention.

*When you watched the news* (*about pA*/*H1N1*), *that risk*, *if you are infected*, *the risk* (*of death*) *was very high*, *how many people died every day*, *it was scary*. (*IM1*)

*This kind of disease is really terrible. Now a lot of people died of cervical cancer*, *a lot*, *such as Anita Mui* (*a Hong Kong celebrity*), *they all died of cervical cancer*, *at so young an age. So*, *early prevention should give some protection to the body*. (*IM19*)

ii. Perceived susceptibility to disease

Parents who were receptive to vaccinating children with optional vaccines usually perceived their child (ren) to be susceptible to those diseases.

*Because when she is admitted to kindergarten*, *if other children have chickenpox she will be very easily infected*. (*IM3*)

In particular, participants perceived greater disease threat if someone they knew was affected.

*Because now cancer is*…*the risk is really high*, *compared to the past*, *there are too many cancers. Many friends around me suffered*. (*IM8*)

iii. Perceived controllability of disease

Participants who perceived low controllability regarding diseases prevention and treatment were more willing to vaccinate their child(ren).

*I once saw somebody had chickenpox*, *the pox were all over the body and the face*, *I was scared to see it. If you do not handle it properly*, *they will have scarring*, *right*? (*IM16*)

*Because particularly girls*, *women*, *will have sexual life*, *and if they have an active sexual life*, *some gynecological diseases*, *I think*, *are unavoidable. Sometimes you totally have no idea why it happened*, *after a long time*, *it will slowly cause lesions*. (*IM7*)

Conversely, parents who believed that VPDs are easily controlled and not so serious usually expressed less or no worry about those diseases, therefore being more inclined to reject the vaccine.

*Chickenpox will not make people die. Just have high fever. If you can handle it well*, *you have no need worry about it*. (*IM15*)

b. Perceived anxiety reduction after vaccination

Fear of the disease is a salient factor motivating parents to vaccinate children. Unsurprisingly, anticipated anxiety reduction after vaccination was a common emotional benefit since physical benefits usually cannot easily be foreseen.

*I think if it is OK*, *better to get the* (*HPV*) *vaccination*, *you can set your mind at rest. Because if there is someone around you suffering from the disease*, *you will worry about it*…*The vaccination will make you feel a little relieved*. (*IM17*)

### Barriers to vaccination

a. Monetary cost

Monetary cost was an important inhibitor for several participants considering optional vaccines for children. A few participants from disadvantaged families rejected all optional vaccines due to the expense.

*Because*, *like our family*, *if you have 2 to 3 children*, *if one vaccine costs two to three hundred dollars*, *the total expense will be a lot. If each extra vaccine costs a few hundred dollars*, *it will be a big burden for family life*. (*IM14*)

High cost was one of the biggest barriers hindering participants who wanted to get HPV vaccination for their daughters.

*Many people hesitate*, *because of the price. It is really expensive. Many people cannot afford it. I know many new immigrants around me and some want to take the vaccine*, *but feel it is too expensive*. (*IM23*)

In contrast, the free provision of mandatory vaccination under CIP was one of the most important reasons for high immunization compliance.

*The government provides* (*vaccines*) *free*, *and it is not bad to be vaccinated*, *so*, *just take it*. (*IM5*)

*The government said* (*vaccinate*) *for prevention*; *then prevent. It is free*! (*IM13*)

b. Concerns about vaccination side effects

Concern about vaccination side effect significantly influenced parental VDM towards “new” vaccines. Several participant mothers didn’t immunize children with pA/H1N1 vaccine due to fear of its potential side effects, and a few participants in particular were concerned about the novel pA/H1N1 vaccine as a new product “hastily” developed in emergency circumstances without sufficient clinical trials.

*We felt like the swine flu vaccine was hastily developed*, *so felt* (*it*) *has side effects. Because the experimental stage was too short*, *it was prematurely distributed*. (*IM5*)

*I felt like it hadn*’*t gone through* (*adequate*) *clinical trial before being provided to people*, *so we were afraid. If a swine flu pandemic emerges again*, (*we*) *may consider taking it*, *because it has now been tested*. (*IM12*)

Several participant mothers felt that the HPV vaccine safety information was ambiguous leaving potentially unknown and long-term side effects.

*Because this vaccine is very new*, *we are not familiar with it. Just heard about it from advertisements*…*For a young child*, *you have to consider*, *are there any side effects*? *Will it influence her future life*, *ah*, *fertility*? (*IM8*)

c. Perceived low efficacy of vaccines

Misconceptions arising from confusing symptoms of influenza and the common cold (“flu”) [28], detracted from the perceived efficacy of influenza vaccination.

*The younger child was vaccinated*, *but after injection*, *within one month*, *she caught cold again. So I think the flu vaccine is useless*. (*IM19*)

Several participant mothers particularly doubted the claimed protection period for HPV vaccine if immunized when young.

*I don*’*t believe it. How come it can protect for several decades*? *It is already good enough to protect 10 or 8 years. Several decades*’ *protection*, *it is impossible*, *isn*’*t it*? (*IM14*)

### Social influences

a. Opinions from significant others

Opinions from significant others (family members, friends, healthcare workers) influenced parental VDM, particularly towards common vaccines.

*I once gave my child a flu shot. My husband disagreed with* (*her having that*) *shot again. Too many injections*, *he said*. (*IM14*)

*One of my customers*, *she asked her friend who was a doctor. That doctor didn*’*t recommend the flu shot. Then*, *the doctors didn*’*t vaccinate their children*, *so why should we take it*? (*IM5*)

b. Follow the crowd

Observing peers’ choice as reference for VDM was common, particularly for new vaccines of perceived uncertain safety and efficacy.

i. Lack of confidence in VDM

A majority of participant mothers tended to wait-and-see what peers’ choices were, often due to lack of confidence in making the right decision. When given the scenario by the interviewer of an optional vaccine, like HPV vaccine, being recommended by government, a few participants still said that they would wait to see what others do.

*It won*’*t increase my confidence*, *because I need to see the crowd around me. I will keep static against the dynamic. If the crowd takes action I will. Otherwise*, *I won*’*t*. (*IM15*)

*Because*…*I don*’*t know anything*, *just life as a housewife*…*I have to ask others*, *to consider the big environment*. (*IM17*)

ii. Information extraction

By observing peers’ choice, parents assimilated relevant information and assessed the safety, effectiveness and necessity of vaccination.

*Well*, *I think it* (*HPV vaccine*) *should be effective*, *otherwise*, *how come so many people go for injection*? (*IM23*)

*In my opinion*, *if other people can take the vaccine*, *it should be fine for us to take it*, *too. Follow the crowd*, *if many of the crowd are vaccinated*, *you have no need to be concerned*. (*IM4*)

iii. Emotional pressure to conform

Several participants reported that they experienced or anticipated anxiety or distress, driving them to conform to group norms.

*If most of others have the shot*, (*that suggests*) *it should be good*….*If others dare not*, *I will also be afraid to. Just like HPV vaccine*, *if most others vaccinate*, *I will also vaccinate*, *otherwise* (*I*) *may have some psychological barriers*, *will be anxious*, *uneasy*. (*IM16*)

The only participant who had vaccinated her child against 2009 pA/H1N1 highlighted the salient role of social influences during parental VDM and the negative consequences.

*To inject or not*? *When you struggle*, *you can*’*t fight the majority*, *so then went to inject*, *because at the beginning many people went for vaccination*…*About 3 months later*, *when I saw the news that a lot of people didn*’*t vaccinate*…*Wow*, *I regretted it so much*. (*IM1*)

…*in (the) next pandemic I will be more thoughtful*, *and not be so rushed into taking action. Seek more information first. During the* (*2009*) *swine flu pandemic*, *I probably was in the first batch vaccinated*, *but now I may be in the last*. (*IM1*)

## Discussion

It was common for respondents to express lack of confidence about choosing whether to vaccinate their child, particularly when the uncertainty and ambiguity surrounding vaccines and VPDs are high. These mothers instead often relied on two powerful psychological principles to help decide: authority and social validation [29]. By following structured expert-based government vaccination programmes mothers can easily choose correctly without struggling with the issue themselves. In the absence of these, conforming to peers’ behaviour utilizes social validation to inform choice. Defined public health programmes like CIP and mandatory school entry requirements removed much uncertainty. All participants accepted CIP vaccines as necessary without explicit consideration, reflecting great trust in medical authority/government [30] and pragmatism (access to schooling). Obedience to authority/government is unusual in vaccine studies [31-33]. Chinese collectivist culture and traditional values respect social order, status hierarchies, and government policies [34]. This effect also manifests in conformity with perceived peer group action to provide reassurance under conditions of risk uncertainty.

Conversely, directives from medical authority/government on optional vaccines are implicit, placing responsibility on parents to decide. Under such circumstances parents’ VDM apparently relies on risk-benefit evaluations [35] for more familiar conditions, such as Chickenpox. However, novel or unfamiliar diseases and vaccines embody uncertain and ambiguous risks and benefits making vaccination decisions more difficult. Consequently, lay responses revert to reliance on heuristics, of which “imitate-the-majority” (bandwagoning [12,15]) appears to be most commonly used [35]. Meanwhile, no participant in our study mentioned herd immunity or adopted the heuristic of “free-riding” [12,36] to guide VDM. By observing peer groups’ action, parents assimilated relevant information and assessed the safety, effectiveness and necessity of vaccination. This will result in many parents adopting a “wait-and-see-approach”. This can significantly impair the value of prophylactic vaccination programmes. Observing peer behaviour before acting probably also reflects conformity and harmony values in Chinese culture.

Numerous previous studies on parents’ disease risk perceptions examined perceived severity of and susceptibility to the diseases [37,38]. Our grounded theory study found that Chinese parents’ perception of VPDs relied on perceived susceptibility and severity, but also emphasized controllability, and anticipation of consequential affective elements as important influences. In particular, VPD-related worry or anxiety was a key factor motivating parents to vaccinate their children. Anticipated anxiety reduction after vaccination is a clear secondary benefit reinforcing vaccination uptake. The Risk-as-feeling hypothesis proposes that negative affect and cognitive risk evaluations are inter-correlated but divergently influencing decision-making [39]. The affect heuristic further argues that feelings, as the emotional aspect of risk perception are as effective, and sometimes better than dispassionate cognitive risk evaluations for informing individual decision-making [40]. The utility model proposes that the primary motivating factor for adopting preventive behavior is resolving the anxiety associated with the threat, rather than the threat itself [41].

Monetary cost was confirmed as a major barrier to vaccination particularly for high-cost HPV vaccine, consistent with a recent systematic review [37]. Here, this was particularly so among study participants who had low household incomes. Vaccination cost emerges as an important contributor to disparities in cervical cancer risk. Routine vaccines are free-of-charge. This is an important facilitator prompting parents to vaccination their children.

Knowledge deficits about vaccines and VPDs appear widespread among new immigrant parents. Constraints on internet access were apparent. Lack of advice about optional vaccines from health professionals was commonly reported. In contrast to the trust in medical authority/government regarding routine vaccines, once direct payment was involved, trust was replaced by suspicion of pecuniary gain, with many parents doubting the motivation behind clinician’s recommendations on optional vaccines, particularly from private clinicians. This contrast is an important finding, illustrating how the public see direct payment distorting the motivations and hence advice from clinicians, and strongly points to a system which is free-at-the-point-of-care as most desirable: money damages trust in health care exchanges. Hence, there is a strong case here for government to issue targeted information and provide vaccination via MCHCs. Empirical studies suggest that people more trusting of formal information (e.g. from media, government, health professional) are more likely to adopt active health-protective behaviors [42,43]. Impaired information communication between health professionals and new immigrant parents probably discourage parents from recommended action and encourage reliance on observing peer behaviour. This is undesirable for several reasons.

Overall, social influence then appears crucial in parental VDM for immigrant Chinese mothers. Three reasons present themselves for the observed high-level of social norm influence in this study. First, study participants relied on limited and mostly informal sources of information. Informal conversation between peers helps to sustain normative group behaviours [44]. Most other information sources involved one-way mass media, which has a tendency towards sensationalism. The limited sources of, and skills to access vaccination-related information restrict information for parental VDM. In this situation, the easiest, most convenient and valid way is to acquire information from observing other’s choices [44]. Secondly, our study participants are all women, and women often show higher levels of group conformity than do men [29]. Third, many participants have low socioeconomic status and may more readily accede to social norms through a greater sense of helplessness than people of higher socioeconomic status [45].

We believe this is the first study on Chinese parental attitudes and decision-making for different childhood and adolescent vaccines. Using qualitative methods we have described parental vaccination decision-making and evidenced several important influences, some consistent with previous studies on European-originating populations but others that are distinctly different. Study limitations include snowball sampling (referrals from original interviewees), which can restrict sample heterogeneity. However, only a small portion of respondents were recruited this way. We particularly selected referred participants purposively on the basis of maximizing sample heterogeneity. Another possible limitation in interviewing mothers, those fathers’ roles may have been under-reported. However, most new immigrants are females and mothers usually are the main decision makers for children’s vaccination in this society (reflected in our present study). In other studies we have performed where we attempted to recruit fathers, they have shown no interest often referring us to their wife as the main health decision makers, reinforcing our belief that the mothers are the key informants to assess. Participants were mostly of low-to-middle-class socioeconomic status and new immigrants tend to take up less skillful jobs in Hong Kong or remain as housewives. Having median monthly household incomes of only 68.6% of that for all households [26], our study sample has characteristics comparable to those of new immigrant mothers generally. Moreover, the findings are consistent with earlier childhood vaccination decision-making studies in Hong Kong [42,46]. So there is good reason to believe that this study presents a valid and reliable picture of the situation faced by many new-immigrant parents in Hong Kong.

### Implications for policy and practice

Mandating childhood vaccines by school entry effectively helps maintain universal vaccination coverage. Future public education and campaigns regarding optional vaccines should clarify necessity and provide explicit guidance. If vaccination is beneficial, there is a case for government provision, and if high uptake is required, vaccinations should be free of charge. Redesigned vaccination record cards could clarify increasingly complicated combination vaccine regimens. These might include both CIP vaccines and others available, with “Must, Should, Could” type recommendations. This would help more parents to make informed decisions and reduce reliance on herd responses. Increasing the channels for vaccination information delivery, concrete vaccination advice from health professionals, particularly public sector clinicians, and school leaflets, plus interactive communications, such as expert-lead community-based health education programmes, would facilitate parental acquisition of more accurate and timely information. Health professionals should attempt to strengthen the decision-makers’ preferred approach to a problem rather than replace it [47] to build trust. Financial cost is an important barrier to parental acceptance of costly optional vaccines, such as chickenpox and HPV vaccine. Policy options should include sliding scale vaccination subsidies to families, or, ideally, including all vaccines in the government-funded CIP.

The Theory of Planned Behaviour (TPB) conceptualizes social influence as “subjective norm” reflecting perceived social pressure to comply from family members, friends, and healthcare providers [48]. However, subjective norms only weakly predict intention [49], while descriptive norms (perceptions of what other people do) are a more substantive predictor of intention [50]. There is medium-to-strong sample-weighted average correlation between descriptive norms and intention [51]. Our findings indicated that descriptive norms appear more influential for parental VDM, particularly towards new vaccines. A reformulation of the social norm concept is needed in future studies of vaccination decision-making.

## Conclusions

In this study we explored how new immigrant mothers from mainland China who had settled in Hong Kong made decisions for children’s vaccination and factors influencing these. Overall, social norms play a key role influencing new immigrant mothers’ VDM, which is the most unique finding from our study. All participants unanimously showed high obedience to public health vaccination programmes, which is remarkably different from findings from studies elsewhere. In part this was a pragmatic decision to ensure their children could enter school uneventfully. Fear of vaccine-targeted diseases rather than dispassionate analysis towards the risk of diseases per se, was a key motivating factor for mothers adopting vaccines for children. Insufficient knowledge about vaccines and targeted diseases, lack of advice from health professionals and, if provided, suspicions regarding the motivations for such advice were common barrier issues. Vaccination cost was a major barrier for some, presumably poorer new immigrant families. Insight gained from this study will help inform healthcare providers develop vaccination communication and policymakers in future vaccination programme, but also have valuable implication for theoretical development on vaccination decision making studies.

## Abbreviations

CIP: Childhood Immunization Programme; DH: Department of Health; MCHCs: Maternal and Child Health Centres; SITs: School Immunization Teams; SARS: Severe Acute Respiratory Syndrome; NCDs: Noncommunicable diseases; HPV: Human papillomavirus; VDM: Vaccination decision making; VPDs: Vaccine-preventable diseases.

## Competing interests

The authors declare that they have no competing interests.

## Authors’ contributions

LDLW contributed to the study design, prepared the research and ethics applications, data collection, data analysis, data interpretation and drafted the manuscript. WWTL contributed to the study design, data analysis, data interpretation, and amended the manuscript. JTW and QYL contributed to data interpretation and manuscript amendment. RF contributed to the study design, data interpretation, and critically amended the manuscript. All authors read and approved the final manuscript.

## Pre-publication history

The pre-publication history for this paper can be accessed here:

http://www.biomedcentral.com/1471-2458/14/133/prepub
